# Optimization of Empirical Antimicrobial Therapy in *Enterobacterales* Bloodstream Infection Using the Extended-Spectrum Beta-Lactamase Prediction Score

**DOI:** 10.3390/antibiotics12061003

**Published:** 2023-06-03

**Authors:** Brian J. Haimerl, Rodrigo Encinas, Julie Ann Justo, Joseph Kohn, P. Brandon Bookstaver, Hana Rac Winders, Majdi N. Al-Hasan

**Affiliations:** 1 Department of Medicine, University of South Carolina School of Medicine, Columbia, SC 29209, USA; brian.haimerl@uscmed.sc.edu (B.J.H.); rodrigo.encinas@uscmed.sc.edu (R.E.); 2 Department of Clinical Pharmacy and Outcomes Sciences, University of South Carolina College of Pharmacy, Columbia, SC 29208, USA; justoj@cop.sc.edu (J.A.J.);; 3 Department of Pharmacy, Prisma Health-Midlands, Columbia, SC 29203, USAhana.winders@prismahealth.org (H.R.W.); 4 Department of Internal Medicine, Division of Infectious Diseases, Prisma Health-Midlands, Columbia, SC 29203, USA

**Keywords:** bacteremia, antibiotics, Gram-negative, *Escherichia coli*, *Klebsiella* species, sepsis, antibiotic resistance

## Abstract

Clinical tools for the prediction of antimicrobial resistance have been derived and validated without examination of their implementation in clinical practice. This study examined the impact of utilization of the extended-spectrum beta-lactamase (ESBL) prediction score on the time to initiation of appropriate antimicrobial therapy for bloodstream infection (BSI). The quasi-experimental cohort study included hospitalized adults with BSI due to ceftriaxone-resistant (CRO-R) *Enterobacterales* at three community hospitals in Columbia, South Carolina, USA before (January 2010 to December 2013) and after (January 2014 to December 2019) implementation of an antimicrobial stewardship intervention. In total, 45 and 101 patients with BSI due to CRO-R *Enterobacterales* were included before and after the intervention, respectively. Overall, the median age was 66 years, 85 (58%) were men, and 86 (59%) had a urinary source of infection. The mean time to appropriate antimicrobial therapy was 78 h before and 46 h after implementation of the antimicrobial stewardship intervention (*p* = 0.04). Application of the ESBL prediction score as part of an antimicrobial stewardship intervention was associated with a significant reduction in time to appropriate antimicrobial therapy in patients with BSI due to CRO-R *Enterobacterales*. Utilization of advanced rapid diagnostics may be necessary for a further reduction in time to appropriate antimicrobial therapy in this population.

## 1. Introduction

Despite major advancements in technology and antimicrobial management throughout the years, bloodstream infection (BSI) remains a leading cause of death in North America and Europe [1]. As antimicrobial resistance rates rise globally, extended-spectrum β-lactamase-producing *Enterobacterales* (ESBL-E) have emerged as one of the most clinically threatening bacteria, causing community-onset infections [2,3,4]. BSIs due to ESBL-E are associated with increased mortality, hospital length of stay, and overall healthcare costs [5,6]. This is particularly relevant as the incidence rate of infections due to ESBL-E has recently increased in the studied community [7,8].

Carbapenems are considered first-line antimicrobial agents for treatment of ESBL-E BSI [9,10]. In the context of the rising rates of antimicrobial resistance and increasing incidence of ESBL-E infection, overutilization of carbapenems is a real concern. Using traditional antimicrobial susceptibility testing, ESBL-E are typically not identified until at least 72 h after growth of bacteria in blood cultures. Early initiation of appropriate antimicrobial therapy has been associated with improved survival and shorter hospital length of stay in patients with Gram-negative BSI [11,12]. Several clinical prediction tools have been derived and validated to identify patients at high risk for BSI due to ESBL-E [13]. However, the utility of these clinical prediction tools is yet to be examined in clinical practice. This quasi-experimental before/after cohort study examined the impact of utilization of the ESBL prediction score [14] on time to initiation of appropriate antimicrobial therapy in patients with BSI due to ceftriaxone-resistant (CRO-R) *Enterobacterales* as part of an antimicrobial stewardship intervention.

## 2. Results

### 2.1. Clinical Characteristics and Microbiology

A total of 146 patients with BSI due to CRO-R *Enterobacterales* were included in this study: 45 before and 101 after implementation of the antimicrobial stewardship intervention. Overall, the median age was 66 years, and 85 (58%) were men. Three-quarters of patients (110; 75%) had community-onset BSI, and the majority (86; 59%) had a urinary source of BSI. Diabetes mellitus was the most common chronic medical condition in this cohort (63; 43%). There were no increased odds of patients being males in the post-intervention compared to the pre-intervention period of the study (odds ratio 1.33; 95% confidence intervals 0.66–2.71). There were no major differences in other baseline demographics and clinical characteristics between the pre- and post-intervention groups of the cohort (Table 1).

**Figure 1:**
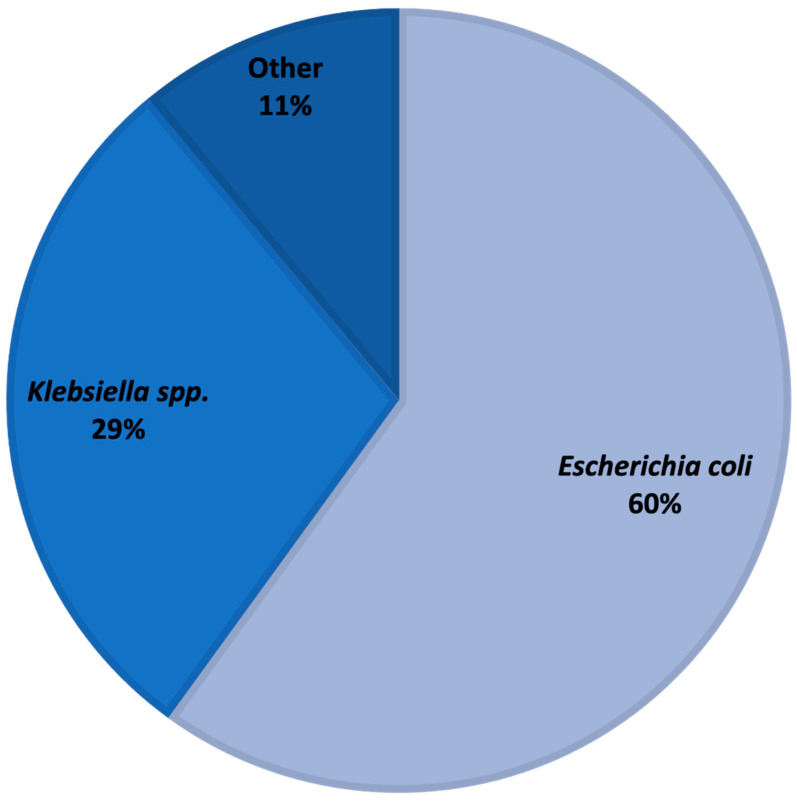
Microbiology of ceftriaxone-resistant *Enterobacterales* bloodstream isolates.

*Escherichia coli* was the most common bloodstream isolate, followed by *Klebsiella* species (Figure 1). ESBL production was the most common known mechanism of ceftriaxone resistance (112; 77%).

### 2.2. Time to Appropriate Antimicrobial Therapy

Overall, the mean time from collection of the index blood culture to the Gram stain and final in vitro antimicrobial susceptibility testing results were 16.8 and 90.7 h, respectively. Empirical antibiotics used before and after the Gram stain results in the pre- and post-intervention periods are summarized in Table 2.

**Figure 2:**
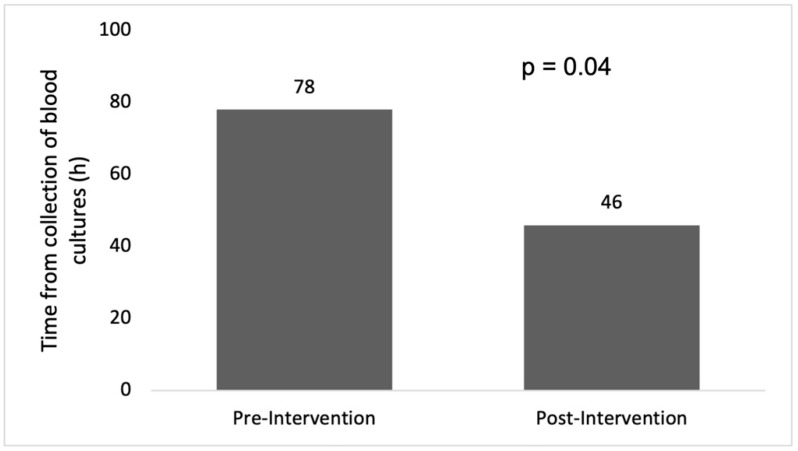
Mean time from collection of blood culture to the initiation of appropriate antimicrobial therapy in the pre- and post-intervention groups.

The mean time to initiation of appropriate antimicrobial therapy was 77.8 h in the pre-intervention group. After implementation of the antimicrobial stewardship intervention using live alerts for positive blood cultures and utilizing the ESBL prediction score, the mean time to initiation of appropriate antimicrobial therapy was 46.3 h (*p* = 0.04), as demonstrated in Figure 2.

## 3. Discussion

### 3.1. Clinical Interpretation of Study Findings

This study demonstrates that use of the ESBL prediction score in the setting of an antimicrobial stewardship intervention can improve empirical antimicrobial therapy in patients with *Enterobacterales* BSI. The ESBL prediction score is determined based on prior antimicrobial use, gastrointestinal or genitourinary procedures, and previous infections or colonization with ESBL-E [14]. In the pre-intervention period, primary healthcare providers initiated empirical antimicrobial therapy based on their clinical experience without using a systematic method for identification of patients at high risk of BSI due to ESBL-E. During that period, the mean time to initiation of appropriate antimicrobial therapy was 78 h. This was a few hours short of the mean time to the final in vitro antimicrobial susceptibility testing results of the bloodstream isolates (91 h). After implementation of the antimicrobial stewardship intervention, with live alerts for positive blood cultures based on the Gram stain results and the application of the ESBL prediction score to guide empirical antimicrobial treatment decisions, the mean time to appropriate therapy was reduced by 32 h. This has significant clinical implications given the potential improvement in clinical outcomes such as mortality and hospital length of stay in association with earlier initiation of appropriate antimicrobial therapy [11,12].

Although this reduction in time to appropriate empirical antimicrobial therapy is both clinically and statistically significant, there appears to be further room for improvement. A mean time to appropriate antimicrobial therapy of 46 h in the post-intervention arm of the study remains less than ideal, particularly in critically ill patients where each hour delay in initiation of appropriate therapy may lead to higher mortality and worse clinical outcomes [15]. The antimicrobial stewardship team receives live alerts for Gram-negative bacilli detected on the Gram stain of blood cultures. On average, these occur 17 h after collection of the index blood cultures. This implies that appropriate antimicrobial therapy is initiated nearly 29 h after the Gram stain alert. There are multiple factors that may contribute to this delay in the initiation of appropriate empirical antimicrobial therapy. The antimicrobial stewardship team reviews a patient’s electronic medical records and calculates the ESBL prediction score soon after the Gram stain report. However, it is common that the antimicrobial stewardship recommendations are made after identification of the bloodstream isolate through a multiplex PCR panel, which takes at least 2 h after the Gram stain results. This allows for de-escalation of the antipseudomonal beta-lactams once *Enterobacterales* BSI is confirmed [16]. There are other logistical delays related to blood culture alerts outside of the routine business hours of the antimicrobial stewardship team and time spent until primary healthcare providers are reached to communicate the recommendations. The use of piperacillin/tazobactam for definitive therapy of BSI due to ESBL-E by some providers, contrary to the antimicrobial stewardship teams’ recommendations, also likely contributed to the delay in initiation of appropriate antimicrobial therapy in this study. The antimicrobial stewardship team recommended empirical treatment with ertapenem in patients with high predicted probability of BSI due to ESBL-E. Since most of the post-intervention period of this study preceded the MERINO trial results [10], these recommendations were not always accepted by the primary team. Some healthcare providers elected to continue empirical therapy with piperacillin/tazobactam rather than switching to carbapenems. Per the study protocol, piperacillin/tazobactam therapy was considered inappropriate for BSI due to ESBL-E, regardless of in vitro antimicrobial susceptibility testing results. This definition was made in priori several years before the MERINO trial results were published [10]. Finally, the ESBL prediction score is not expected to identify all patients with BSI due to ESBL-E as per the score’s previously published performance characteristics [13,14].

### 3.2. Potential Applications of the ESBL Prediction Score

The ESBL prediction score represents a quick, simple, and inexpensive tool for optimization of empirical antimicrobial therapy in patients with *Enterobacterales* BSI. The ESBL prediction score may be used in conjunction with an existing antimicrobial stewardship intervention for Gram-negative BSI, as in the current study. Utilization of the ESBL prediction score by antimicrobial stewardship teams would likely provide maximum benefit as the antimicrobial resistance risk prediction would be coupled with vast knowledge and clinical experience in antimicrobial management. In resource-limited settings where antimicrobial stewardship monitoring of positive blood cultures may not be feasible, the ESBL prediction score may be used directly by the primary clinical providers. This may be implemented after validation of the score at the local site as part of an institutional management guideline that utilizes the ESBL prediction score to streamline empirical antimicrobial therapy for Gram-negative BSI. Providing clinicians with access to the ESBL prediction score through a printed or online guidebook or an interactive tool on a mobile device application may improve utilization of the score.

### 3.3. Clinical Prediction Tools versus Advanced Rapid Diagnostics

A faster initiation of appropriate antimicrobial therapy is the ultimate goal in the management of patients with BSI. As demonstrated in the current study, utilization of clinical tools for the prediction of antimicrobial resistance can help approach that goal. However, advanced rapid diagnostics are likely required to achieve that target. The BioFire^®^ FilmArray blood culture identification panel 2 (BCID2) is a novel multiplex PCR assay which includes nine additional microbial targets and seven additional antimicrobial resistance genes in comparison to its predecessor [17]. One of the additional antimicrobial resistance genes is CTX-M, which accounts for nearly 80% of detected ESBL genes in North America and Europe [18,19]. BCID2 has demonstrated significantly shorter turnaround times for organism identification in comparison to traditional methods [20]. Hypothetically, it has the potential to reduce time to appropriate antimicrobial therapy to <24 h in the majority of patients with BSI due to CRO-R *Enterobacterales* [20]. The downside of using BCID2 is missing less common ESBL genes and other mechanisms of CRO-R in *Enterobacterales*, such as AmpC production. The Accelerate Pheno^®^ system (Pheno) is another novel rapid diagnostic test that can provide organism identification within 90 minutes and phenotypic antimicrobial susceptibility testing results within 7 h of the Gram stain report [21]. It, too, has demonstrated a decrease in time to effective antimicrobial therapy, a measure of critical importance in patients with *Enterobacterales* BSI [21]. However, utilization of these rapid diagnostic tests for further improvement in antimicrobial management comes at a considerable cost to patients and healthcare systems. There are, however, some inexpensive rapid diagnostics that can be performed directly from a positive blood culture. Lateral flow immunoassays are cost-effective, phenotypic tests which have demonstrated the ability to identify resistant organisms in under 2 h [22]. Colormetric assays are another option for rapid, cost-effective identification, and these have the added advantage of being able to identify non-CTX-M EBSL-producing organisms [23]. Furthermore, the European Committee on Antimicrobial Susceptibility Testing has developed a rapid disk diffusion method for rapid antimicrobial susceptibility testing. It has been demonstrated as effective in identifying ESBL-producing organisms as early as 4 h after positive blood culture and can be used even in resource-limited settings [24].

### 3.4. Strengths and Limitations

This study applied a validated ESBL prediction score to clinical practice and evaluated its efficacy in comparison to the standard methods at the time. To our knowledge, this is the first study to provide a real-life evaluation of any clinical tool for the prediction of antimicrobial resistance in hospital practice.

Limitations of this study include a patient population from a single healthcare system which is affected by local demographics, epidemiology, and antimicrobial resistance patterns. The results from this study may not be entirely applicable to other geographical locations with considerably higher ESBL-production rates among *Enterobacterales* bloodstream isolates. Additionally, due to this study’s focus on patients with CRO-R *Enterobacterales* BSI, it has a relatively small sample size of 146. The current study used time to appropriate antimicrobial therapy as the primary outcome of patients with BSI, which has been associated with clinical outcomes such as mortality and hospital length of stay [11,12]. The study lacked adequate power to directly examine these clinical outcomes, particularly since the majority of included patients were not critically ill enough to require admission to intensive care units. An additional limitation of this study is the fact that ESBL prediction scores were only calculated after notification of a positive blood culture. It would be useful to examine the utilization of the score at the time of blood culture collection and initiation of empirical antibiotic therapy in future studies.

## 4. Methods

### 4.1. Setting

The study was conducted at three Prisma Health-Midlands Hospitals in South Carolina. These represent a community-teaching hospital and two community hospitals that serve the population in the Midlands region of South Carolina and receive referrals from nearby areas in the state. The three hospitals combined together provide over 1000 licensed beds and provide healthcare for a wide variety of medical and surgical subspecialties. The study was approved by the Institutional Review Board at Prisma Health (study identification number 1852677-2, amendment 7, approval date 27 April 2022). A waiver of informed consent was granted.

### 4.2. Case Ascertainment

All hospitalized adults 18 years of age or older with monomicrobial BSI due to CRO-R *Enterobacterales* at Prisma Health-Midlands Hospitals in South Carolina from 1 January 2010 to 31 December 2019 were included (*n* = 146). Recurrent episodes of *Enterobacterales* BSI during the study period were excluded.

### 4.3. Microbiology Techniques

Blood cultures were processed using Clinical and Laboratory Standards Institute (CLSI) techniques. In vitro antimicrobial susceptibilities were determined via the VITEK^®^ 2 system using the CLSI criteria. Bloodstream isolates that were non-susceptible in vitro to any tested third-generation cephalosporin were screened for ESBL production using a disk diffusion method with cefotaxime/clavulanate combination disks. BioFire^®^ blood culture identification multiplex PCR panel I (BCID1) was introduced in the post-intervention period. The panel allows for the identification of bloodstream isolates within 2 h of the Gram stain results. It also identifies common genes for carbapenemases (e.g., KPC), but not ESBL genes (e.g., CTX-M).

### 4.4. Antimicrobial Stewardship Intervention

**Figure 3:**
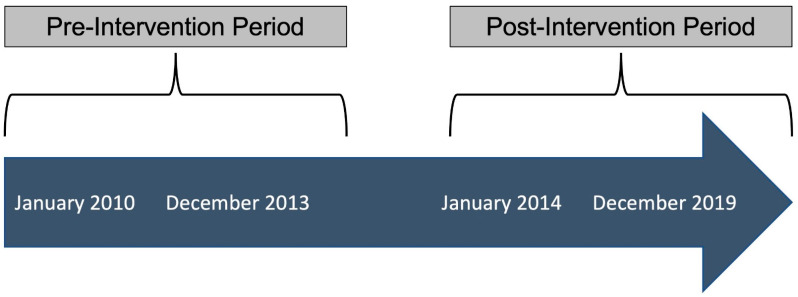
Timeline of antimicrobial stewardship intervention implementation.

An antimicrobial stewardship intervention was implemented on 1 January 2014 consisting of real-time alerts for positive blood cultures via a Gram stain. The pre-intervention period was 1 January 2010 through to 31 December 2013, and the post-intervention period followed from 1 January 2014 through to 31 December 2019 (Figure 3). At the time of an alert, the antimicrobial stewardship team applied the ESBL prediction score. The antimicrobial stewardship team made recommendations on empirical antimicrobial therapy based on the predicted probability of BSI due to ESBL-E using the ESBL prediction score before conventional in vitro antimicrobial susceptibility testing results were available. The antimicrobial regimen was further revised, if needed, based on the results of the conventional in vitro antimicrobial susceptibility testing results.

The ESBL prediction score, a clinical tool that utilizes three criteria to predict the risk of ESBL-E BSI, was used for risk stratification. Variables included in the ESBL prediction score were outpatient genitourinary/gastrointestinal procedure within 30 days of BSI (1 point), β-lactam or fluoroquinolone use within 90 days (1 point for 1 course or 3 points for multiple courses), and prior infection/colonization with an ESBL-E within past 12 months (4 points). Patients with an ESBL prediction score < 1 were deemed at low risk of BSI due to ESBL-E. The antimicrobial stewardship team recommended empirical treatment with ceftriaxone for most *Enterobacterales*, including *E. coli*, *Klebsiella* species, and *P. mirabilis*. Cefepime was recommended for *Enterobacter cloacae* and other potentially ampC-producing *Enterobacterales*. Patients with a calculated ESBL prediction score ≥ 3 were considered at high risk of BSI due to ESBL-E. The recommendations were to start empirical therapy with ertapenem for these patients. A calculated ESBL prediction score of 1–2 represented a moderate risk of BSI due to ESBL-E. Treatment recommendations were determined for these patients based on acute severity of illness. Ertapenem was recommended in critically ill patients. Carbapenems were deferred in non-critically ill patients with moderate risk of BSI due to ESBL-E [14].

**Figure 4:**
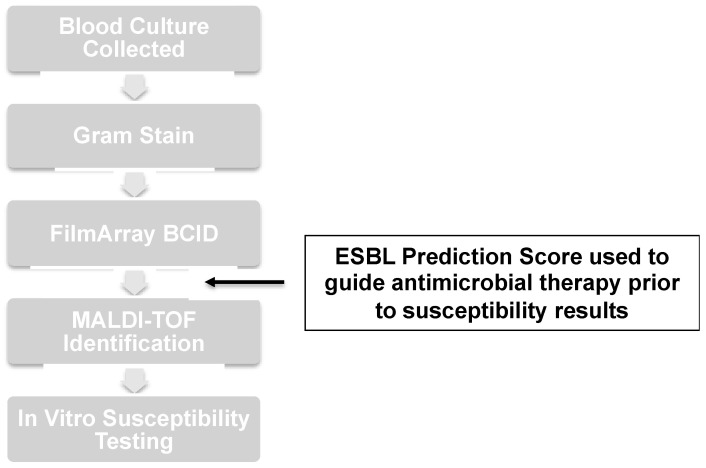
Application of ESBL prediction score in management of *Enterobacterales* BSI.

Antimicrobial stewardship recommendations were communicated with the primary healthcare providers promptly after bacterial identification of bloodstream isolates through a multiplex-PCR panel (Figure 4). Notably, the antimicrobial stewardship team operated during weekday business hours (Monday through Friday from 8:00 am to 4:30 pm). The ESBL prediction score and clinical decision algorithm were available to healthcare providers in print and via the Prisma Health internal website. This access allowed clinicians to use the ESBL prediction score on their own to initiate empirical antimicrobial therapy when the antimicrobial stewardship team was not available. A free interactive mobile device application was also made available to primary providers to stratify the risk of BSI due to ESBL-E [https://firstline.org; accessed on 28 April 2023].

The overall impact of the antimicrobial stewardship intervention on patients with Gram-negative BSI was previously described after 18 months of implementation [16]. However, the impact of the intervention on CRO-R *Enterobacerales* BSI was unclear due to the small number of these isolates over a relatively short period of time. This study was performed to evaluate the impact of the intervention specifically on BSI due to CRO-R *Enterobacterales* after 6 years of implementation to increase the number of these bloodstream isolates and allow for meaningful comparisons.

### 4.5. Outcomes and Statistical Analysis

The primary outcome of this study was the difference in mean time to appropriate antimicrobial therapy in the pre- and post-intervention periods of the study. The time to appropriate antimicrobial therapy was calculated as the time from the collection of the index blood culture until the initiation of the appropriate antimicrobial therapy. Antimicrobial therapy was considered appropriate if the bloodstream isolate was susceptible in vitro to one or more antimicrobial agents in the empirical regimen using the Clinical Laboratory Standards Institute criteria. For ESBL-E BSI, only carbapenems (ertapenem, meropenem, or imipenem/cilastatin) were considered appropriate from the beta-lactam antimicrobial class. Penicillins and cephalosporins, including piperacillin/tazobactam and cefepime, were considered inappropriate for treatment of ESBL-E BSI, regardless of in vitro antimicrobial susceptibility testing results [11]. All study definitions were determined in priori.

A Student’s *t*-test was performed to examine the difference in the mean time to appropriate empirical antimicrobial therapy before and after the implementation of the antimicrobial stewardship intervention. The level of significance for statistical testing was defined as *p* < 0.05. JMP Pro (version 16.0; SAS Institute, Cary, NC, USA) was used for all statistical analysis.

## 5. Conclusions

Application of the ESBL prediction score as part of an antimicrobial stewardship intervention was associated with a significant reduction in time to appropriate antimicrobial therapy in patients with BSI due to CRO-R *Enterobacterales*. Utilization of more advanced rapid diagnostics such as multiplex PCRs for the detection of CTX m and other common ESBL genes or novel methods for rapid phenotypic antimicrobial susceptibility testing may be necessary for a further reduction in time to appropriate antimicrobial therapy in this population.

## Figures and Tables

**Table 1:** Demographics and clinical characteristics of patients with ceftriaxone-resistant *Enterobacterales* bloodstream infection.

Variable	Total (*n* = 146)	Pre-Intervention (*n* = 45)	Post-Intervention (*n* = 101)	*p*-Value
Median age (IQR) in years	66 (51–75)	68 (50–76)	63 (51–75)	0.27
Male sex	85 (58)	24 (53)	61 (60)	0.42
Hospital-onset BSI	37 (25)	8 (18)	29 (29)	0.16
Residence at SNF	33 (23)	12 (27)	21 (21)	0.43
Urinary source of BSI	86 (59)	26 (58)	60 (59)	0.85
Diabetes mellitus	63 (43)	17 (38)	46 (46)	0.38
End-stage renal disease	13 (9)	5 (11)	8 (8)	0.53
Liver cirrhosis	4 (3)	3 (7)	1 (1)	0.09
Cancer	24 (16)	9 (20)	15 (15)	0.44
Immune compromised	18 (12)	4 (9)	14 (14)	0.40
ICU admission	43 (29)	14 (31)	29 (29)	0.77

Data are shown as numbers (%) unless otherwise specified. IQR: interquartile range; BSI: bloodstream infection; SNF: skilled nursing facility; ICU: intensive care unit.

**Table 2:** Empirical antibiotics used before and after the Gram stain results in the pre- and post-intervention periods of the study.

Antibiotic	Pre-Intervention Period (*n* = 45)		Post-Intervention Period (*n* = 101)	
	Before Gram Stain	After Gram Stain	Before Gram Stain	After Gram Stain
Ampicillin/sulbactam	0 (0)	0 (0)	3 (3)	0 (0)
Ceftriaxone	9 (20)	8 (18)	28 (28)	5 (5)
Cefepime	4 (9)	3 (7)	29 (29)	14 (14)
Piperacillin/tazobactam	14 (31)	12 (27)	26 (26)	12 (12)
Ertapenem	3 (7)	7 (16)	7 (7)	52 (52)
Meropenem	8 (18)	9 (20)	6 (6)	14 (14)
Fluoroquinolones *	6 (13)	3 (7)	1 (1)	4 (4)
Gentamicin	1 (2)	3 (7)	1 (1)	0 (0)

Data are shown as numbers (%). * Ciprofloxacin or levofloxacin.

## Data Availability

Data are available upon request.

## Abbreviations

BSI—bloodstream infection; ESBL—extended-spectrum β-lactamase; CRO-R—ceftriaxone-resistant; ESBL-E—extended-spectrum β-lactamase-producing *Enterobacterales*; BCID—BioFire FilmArray blood culture identification panel; CLSI—Clinical and Laboratory Standards Institute; PCR—Polymerase Chain Reaction.
